# Supplementary material for: Heat Stress and Plant–Biotic Interactions: Advances and Perspectives
Source: Plants (Basel). 2024 Jul 23;13(15):2022. doi: 10.3390/plants13152022 (PMC11313874; doi:10.3390/plants13152022)
Supplement: Supplementary file 1 [file plants-13-02022-s001.zip › plants-3070704-supplementary.pdf]

Supplementary Information

Review

Heat Stress and Plant–Biotic Interactions: Advances and Perspectives

Rahul Mahadev Shelake [1,*,†], Sopan Ganpatrao Wagh [2,*,†], Akshay Milind Patil [3], Jan Červený [2], Rajesh Ramdas Waghunde [4] and Jae-Yean Kim [1,5,6,*]

[1] Division of Applied Life Science (BK21 Four Program), Plant Molecular Biology and Biotechnology Research Center, Gyeongsang National University, Jinju 52828, Republic of Korea
[2] Global Change Research Institute, Czech Academy of Sciences, Brno 60300, Czech Republic; cerveny.j@czechglobe.cz
[3] Cotton Improvement Project, Mahatma Phule Krishi Vidyapeeth (MPKV), Rahuri 413722, India; akshaypatilbiotech@gmail.com
[4] Department of Plant Pathology, College of Agriculture, Navsari Agricultural University, Bharuch 392012, India; rajeshpatho191@nau.in
[5] Division of Life Science, Gyeongsang National University, Jinju 52828, Republic of Korea
[6] Nulla Bio Inc., Jinju 52828, Republic of Korea
* Correspondence: rahultnau@gmail.com or rahulms@gnu.ac.kr (R.M.S.); wagh.s@czechglobe.cz or swagh.gene@gmail.com (S.G.W.); kimjy@gnu.ac.kr (J.-Y.K.); Tel.: +82-55-772-1361 (J.-Y.K.)
† These authors contributed equally to this work.

Supplementary Table S1. Significant yield losses caused by the impact of combined biotic and abiotic stressors in agriculture. The approximate percentages vary by region, climate conditions, and management practices. Biotic stressors include pathogens like fungi, bacteria, viruses, and pests such as insects and nematodes. Abiotic stressors encompass environmental factors like extreme temperatures, water scarcity (drought), excessive rainfall, and soil conditions.

Commodity	Estimated Annual Yield Loss (%)	Biotic Stressors	Abiotic Stressors
Wheat	40-50%	Diseases (rusts, blights), pests (aphids)	Drought, heat stress, soil salinity
Rice	30-40%	Diseases (blast, bacterial blight), pests	Flooding, drought, high temperatures
Maize (Corn)	30-60%	Diseases (leaf blights, stalk rots), pests	Drought, heat stress, soil nutrient deficiencies
Soybean	30-50%	Diseases (soybean rust, root rot), pests	Drought, heat stress
Potato	40-70%	Diseases (late blight, bacterial wilt), pests	Drought, heat stress
Cotton	20-30%	Pests (bollworms, aphids), diseases	Drought, heat stress

References

Wheat:

- Savary, S.; Willocquet, L.; Pethybridge, S.J.; Esker, P.; Mcroberts, N.; Nelson, A. The Global Burden of Pathogens and Pests on Major Food Crops. *Nat. Ecol Evol* **2019**, 3, 430–439. doi:10.1038/s41559-018-0793-y
- Asseng, S.; Ewert, F.; Martre, P.; Rötter, R.P.; Lobell, D.; Cammarano, D.; Kimball, B.; Ottman, M.; Wall, G.; White, J.W. Rising Temperatures Reduce Global Wheat Production. *Nat. Clim. Change* **2015**, 5, 143. doi:10.1038/nclimate2470

Rice:

- Savary, S.; Willocquet, L.; Pethybridge, S.J.; Esker, P.; Mcroberts, N.; Nelson, A. The Global Burden of Pathogens and Pests on Major Food Crops. *Nat. Ecol Evol* **2019**, 3, 430–439. doi:10.1038/s41559-018-0793-y
- Peng, S.; Huang, J.; Sheehy, J.E.; Laza, R.C.; Visperas, R.M.; Zhong, X.; Centenso, G.S.; Khush, G.S.; Cassman, K.G. Rice Yields Decline with Higher Night Temperature from Global Warming. *Proc Natl Acad Sci USA* **2004**, 101, 9971–9975. doi:10.1073/pnas.0403720101

Maize (Corn):

- Oerke, E.C. Crop Losses to Pests. *J Agric Sci* **2006**, 144, 31–43. doi:10.1017/S0021859605005708
- Lobell, D.B.; Schlenker, W.; Costa-Roberts, J. Climate Trends and Global Crop Production Since 1980. *Science* **2011**, 333, 616–620. doi:10.1126/science.1204531

Soybean:

- Hartman, G.L.; West, E.D.; Herman, T.K. Crops that feed the World 2. Soybean—Worldwide Production, Use, and Constraints Caused by Pathogens and Pests. *Food Secur* **2011**, 3, 5–17. doi: 10.1007/s12571-010-0108-x
- Battisti, D.S.; Naylor, R.L. Historical Warnings of Future Food Insecurity with Unprecedented Seasonal Heat. *Science* **2009**, 323, 240–244. doi:10.1126/science.1164363

Potato:

- Oerke, E.C. Crop Losses to Pests. *J Agric Sci* **2006**, 144, 31–43. doi:10.1017/S0021859605005708
- Raymundo, R.; Asseng, S.; Robertson, R.; Petsakos, A.; Hoogenboom, G.; Quiroz, R.; Hareau, G.; Wolf, J. Climate Change Impact on Global Potato Production. *Eur J Agron* **2018**, 100, 87–98. doi:10.1016/j.eja.2017.11.008

Cotton:

- Oerke, E.C. Crop Losses to Pests. *J Agric Sci* **2006**, 144, 31–43. doi:10.1017/S0021859605005708
- Reddy, K.R.; Hodges, H.F.; McKinion, J.M.; Wall, G.W. Temperature Effects on Pima Cotton Growth and Development. *Agron J* **1992**, 84, 237–243. doi:10.2134/agronj1995.00021962008700050010x
